# Estimating sequencing error rates using families

**DOI:** 10.1186/s13040-021-00259-6

**Published:** 2021-04-23

**Authors:** Kelley Paskov, Jae-Yoon Jung, Brianna Chrisman, Nate T. Stockham, Peter Washington, Maya Varma, Min Woo Sun, Dennis P. Wall

**Affiliations:** 1 Department of Biomedical Data Science, Stanford University, Stanford, CA USA; 2 Department of Pediatrics (Systems Medicine), Stanford University, Stanford, CA USA; 3 Department of Bioengineering, Stanford University, Stanford, CA USA; 4 Department of Neuroscience, Stanford University, Stanford, CA USA; 5 Department of Computer Science, Stanford University, Stanford, CA USA

**Keywords:** Sequencing error, Whole-genome sequencing, Whole-exome sequencing, Microarray, Families

## Abstract

**Background:**

As next-generation sequencing technologies make their way into the clinic, knowledge of their error rates is essential if they are to be used to guide patient care. However, sequencing platforms and variant-calling pipelines are continuously evolving, making it difficult to accurately quantify error rates for the particular combination of assay and software parameters used on each sample. Family data provide a unique opportunity for estimating sequencing error rates since it allows us to observe a fraction of sequencing errors as Mendelian errors in the family, which we can then use to produce genome-wide error estimates for each sample.

**Results:**

We introduce a method that uses Mendelian errors in sequencing data to make highly granular per-sample estimates of precision and recall for any set of variant calls, regardless of sequencing platform or calling methodology. We validate the accuracy of our estimates using monozygotic twins, and we use a set of monozygotic quadruplets to show that our predictions closely match the consensus method. We demonstrate our method’s versatility by estimating sequencing error rates for whole genome sequencing, whole exome sequencing, and microarray datasets, and we highlight its sensitivity by quantifying performance increases between different versions of the GATK variant-calling pipeline. We then use our method to demonstrate that: 1) Sequencing error rates between samples in the same dataset can vary by over an order of magnitude. 2) Variant calling performance decreases substantially in low-complexity regions of the genome. 3) Variant calling performance in whole exome sequencing data decreases with distance from the nearest target region. 4) Variant calls from lymphoblastoid cell lines can be as accurate as those from whole blood. 5) Whole-genome sequencing can attain microarray-level precision and recall at disease-associated SNV sites.

**Conclusion:**

Genotype datasets from families are powerful resources that can be used to make fine-grained estimates of sequencing error for any sequencing platform and variant-calling methodology.

## Background

In order to responsibly use the results of genetic testing in patient treatment, clinicians need good estimates of the likelihood of false positive and false negative test results [1]. This is a major obstacle for moving next generation sequencing methods into the clinic since variant calls are highly dependent not only upon the details of the sequencing assay itself, but also on the software pipeline used to analyze the data [2]. While best-practices have been established [3], software pipelines are continuously evolving, with new versions released every few years. This makes it difficult to estimate error rates for the exact combination of sequencing platform and software pipeline used to generate data for each patient.

The primary method for estimating the error rate of a sequencing method is replication [4]. The same individual is sequenced multiple times, often using different sequencing platforms and variant calling pipelines in order to produce a set of consensus calls. These consensus calls are then used as the ground truth in order to evaluate a new sequencing platform or software pipeline. This method has been used by the genome-in-a-bottle (GIAB) consortium [5] and Illumina’s platinum genomes project [6] to produce publicly-available “gold-standard” calls that have been widely used to benchmark new methods and algorithms. The consensus method has been used to quantify the performance of sequencing platforms [7], aligners [8, 9], and variant calling algorithms [10, 11].

Consensus methods have several limitations. First, sequencing the same individual multiple times is expensive, so sometimes computational replicates (running different analysis pipelines on the same raw sequencing data) or technical replicates (sequencing the same sample) are used in place of true biological replicates (sequencing multiple samples from the same individual). Using replicates from different points in the sequencing process can cause replicates to share errors, which in turn produces erroneous consensus calls. For example, because computational replicates all work off of the same raw reads, they will be susceptible to the same PCR-amplification errors, when true biological replicates would not.

Consensus methods are also sensitive to the number of replicates conducted per sample. Sometimes as few as two or three replicates are used, in which case consensus methods can produce an estimate of precision, but struggle to estimate recall. This is because with a small number of replicates, calls where all methods agree are considered true positives, but calls where methods disagree are more difficult to classify. An estimate of recall requires knowledge of the number of false negatives, which is only available if you have enough replicates to identify which call is correct when replicates disagree.

Finally, consensus methods focus on comparing replicates of a single individual, or at best a handful of individuals, making it difficult to study error rate variability from individual to individual or sample to sample. Inter-individual variability in sequencing error has been observed in the HLA region due to mapping bias, where reads containing variants map less accurately than reads without variants, resulting in erroneous calls occurring more frequently in individuals with non-reference genotypes [12]. Furthermore, differences in sample preparation have also been shown to affect sequencing error rates [13, 14]. Sample-specific error models have been shown to improve sensitivity and specificity of variant calling in tumor samples [15], suggesting that sequencing error rates may vary considerably from sample to sample. Our inability to quantify variability in error rates from sample to sample makes it difficult to extrapolate error rates estimated from GIAB reference material to patient data.

Using sequencing data from parents and their children provides a unique opportunity to address these problems. Since children share 50% of their genetic material with each of their parents, sequencing data from families is similar to a biological replicate, allowing a fraction of the sequencing errors present in the family to be observed as Mendelian errors. Of course, not all sequencing errors result in Mendelian errors, so methods have been developed to use counts of Mendelian errors to predict the total number of sequencing errors in a family [16] and to identify quality control metrics that are indicative of sequencing errors [17]. Here, we extend these approaches to produce estimates of precision and recall at heterozygous and homozygous alternate sites for each individual in the family. Our method uses Poisson regression to model the observed frequencies of different Mendelian errors to estimate error rates and can be applied to any sequencing pipeline. We validate the accuracy of our error estimates using identical twins. We then use a set of identical quadruplets to show that our family-based method produces estimates of precision and recall that closely match those produced by the consensus method. We then apply our method to five large sequencing datasets, allowing us to study individual-level variability in precision and recall across thousands of individuals sequenced by whole-genome, whole-exome and microarray platforms. We show that by using our method within family data, we can more effectively detect errors than current approaches.

## Results

### Estimating sequencing error rates

**Fig. 1:**
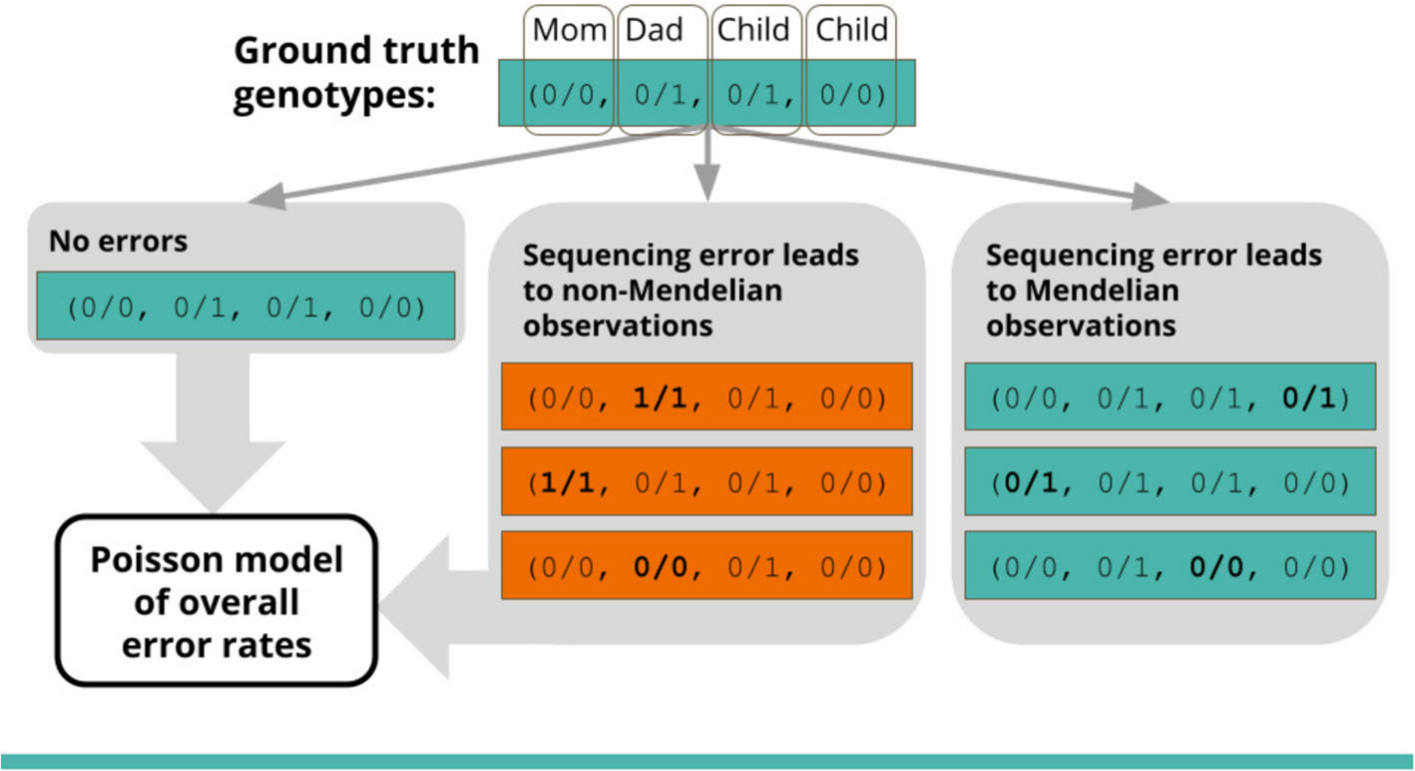
Some sequencing errors produce non-Mendelian observations in the family. By modelling the frequency of these non-Mendelian observations, as compared to the frequency of neighboring Mendelian observations, we can estimate the overall sequencing error rate for each individual

Family data allows us to directly detect some, but not all, sequencing errors because they produce non-Mendelian observations in the family, as shown in Fig. 1. By using Poisson regression to model the frequency of these non-Mendelian observations as compared to the frequency of their neighboring Mendelian-consistent observations, we can estimate the precision and recall of variant calls at both heterozygous and homozygous alternate sites for each individual in a family. Our method uses familial relatedness to produce estimates of the overall variant call error rate for each sample, even though many errors do not result in non-Mendelian family genotypes and are therefore not directly observable. More detail along with a derivation of our model is given in “Methods” section.

### Validating sequencing error rate estimates using monozygotic twins

We begin by validating our family-based error-estimation method using monozygotic twins. Sequencing data from monozygotic twins do not necessarily provide perfect ground truth genotype information, because when twins exhibit different genotypes at a site, we have no way of knowing which twin’s genotype is correct and which is the result of a sequencing error. However, we can still use monozygotic twins to validate our error estimates by comparing the number of sites where the twins have mismatched genotype calls to the number of such sites we would expect given our error estimates.

We use monozygotic twins from three different datasets to validate our method, including one whole-genome sequencing dataset (iHART WGS), one whole-exome sequencing dataset (SPARK WES), and one microarray dataset (SPARK Array).

**Fig. 2:**
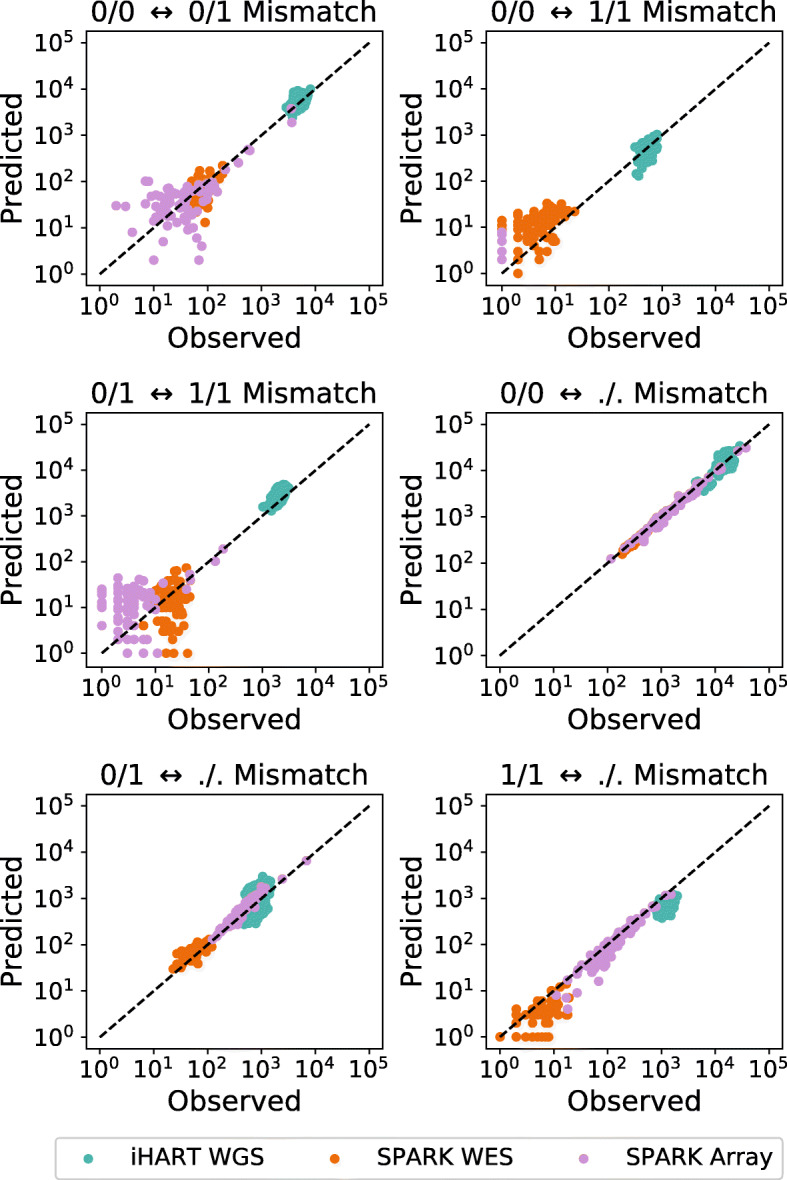
We validate our method using monozygotic twins. Using our estimated error rates, we predict the number of sites that will differ between identical twins. We then compare our predictions to the observed counts. We see that the predicted number of mismatches closely matches the observed counts

Figure 2 compares the observed genotype mismatches for each pair of twins to the predicted number of mismatches, given our error estimates. Differing sequencing error rates and SNP densities between sequencing platforms cause the number of mismatched genotypes to vary over five orders of magnitude. Our method produces accurate predictions across this wide range.

### Validating sequencing error rate estimates with the consensus method

**Fig. 3:**
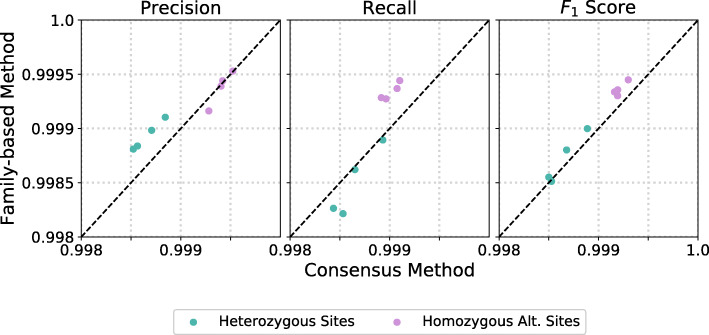
Using a set of monozygotic quadruplets, we compare our family-based error estimation method to the consensus method. We see that our family-based estimates closely match consensus estimates

Next, we validate our family-based error rate estimates by comparing them to the consensus method using a set of identical quadruplets from the iHART dataset. To produce consensus estimates, we use all sites where three or more of the quadruplets have the same variant call, and we consider the consensus call to be the *ground truth* genotype. We then calculate precision, recall, and *F*_1_ score for each quadruplet and compare these values to those produced by our family-based method. Figure 3 shows that our family-based method and the widely-used consensus method produce very similar results.

### Comparing variant calling performance across sequencing platforms

Next, we use our error estimation method to look at both inter-dataset and intra-dataset error rate variability across samples from a variety of sequencing platforms. We use sequencing data from five different sequencing datasets to evaluate our method. These include two whole-genome sequencing datasets (iHART WGS and SSC WGS), one whole-exome sequencing dataset (SPARK WES), and two microarray datasets (iHART Array and SPARK Array).

Sequencing platforms such as microarrays or WES can only identify variants within particular genomic regions (target regions for WES and target sites for microarrays), while WGS identifies variants anywhere in the genome. Our precision and recall measurements take these restrictions into account, so precision and recall for microarray samples are evaluated only on sites targeted by the microarray, while for WGS samples, they are evaluated using all variants in the genome.

**Fig. 4:**
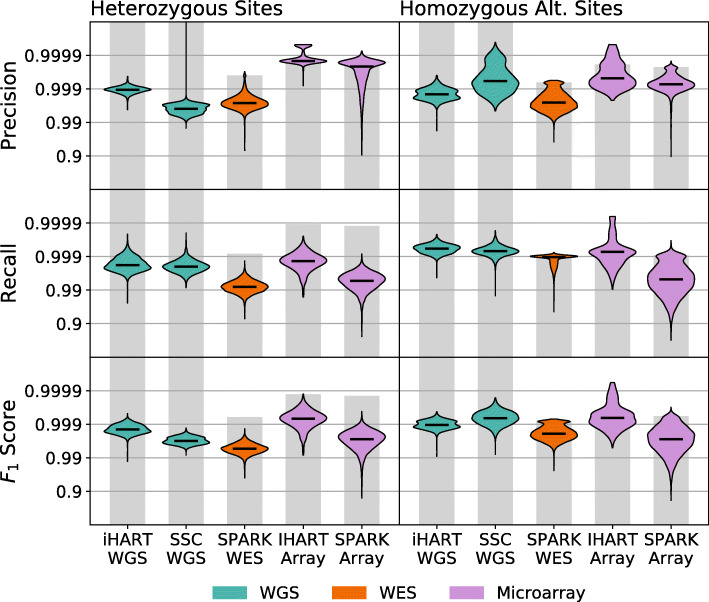
Error rates exhibit inter- and intra-dataset variability. The violin plots show the distribution of per-sample precision and recall measurements. The top panels show precision and recall at heterozygous sites. The bottom panels show precision and recall at homozygous alternate sites. The grey bars indicate the most extreme precision and recall measurements that can be supported by the SNP density of the dataset. Unsurprisingly, precision and recall vary across sequencing datasets. However, we noticed unexpected sample-level variability in precision and recall within datasets, indicating that samples sequenced on the same platform and analyzed with the same software pipeline may have dramatically different error rates

Figure 4 shows per-sample distributions of precision, recall, and *F*_1_ score for each dataset. Our algorithm produces these estimates for both heterozygous sites and homozygous alternate sites. We see immediately that precision, recall, and *F*_1_ score all vary dramatically between samples within the same dataset, meaning that even if samples are sequenced using the same platform and processed with the same variant calling pipeline, precision and recall may still vary across an order of magnitude. This indicates that many samples are required to gain an accurate picture of the performance of a sequencing pipeline. The accuracy of microarray variant calls in particular seems to have the largest per-sample variance.

### GATK v3.2 vs GATK v3.4

**Fig. 5:**
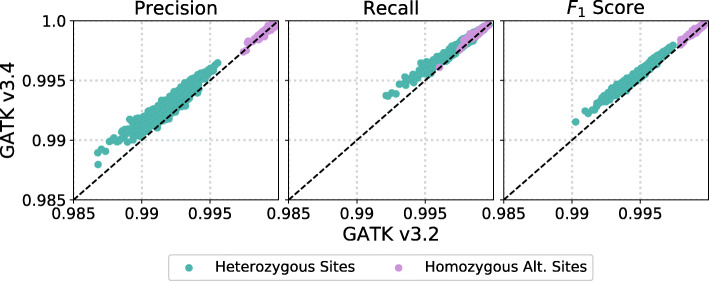
Our method is sensitive enough to detect differences in performance between different software versions of the GATK variant calling pipeline. We compare precision and recall for 965 samples from iHART using GATKv3.2 and GATKv3.4, using the same raw reads. We see an improvement in both precision and recall at heterozygous sites, and a slight improvement in recall at homozygous alternate sites

Next, we demonstrate that our method is sensitive enough to quantify improvements in variant calling pipelines from one version to another. GATKv3.2 and GATKv3.4 are two versions of the same variant calling software pipeline. In Fig. 5, we compare variant calling error rates between these versions. Variants were called on the same set of samples from iHART, using the same read alignments.

GATKv3.4 improves precision and recall as compared to GATKv3.2 with a median decrease in false discovery rate (1-precision) of 15% and 10% for heterozygous and homozygous alternate sites respectively and a median decrease in false negative rate (1-recall) of 15% and 14% for heterozygous and homozygous alternate sites respectively.

### Human reference GRCh37 vs GRCh38

During the variant calling process, reads are first aligned to the human reference genome before variants are called. Reads containing variants as compared to the human reference map less well than reads with no variants, and this mapping bias has been shown to result in poorer variant calling performance at sites where an individual differs from the reference [12]. These results suggest that as the human reference improves, variant calling performance should also improve.

**Fig. 6:**
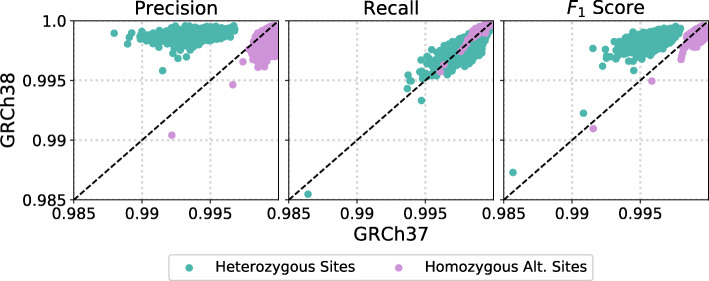
We compare GATK’s variant calling performance using different versions of the human reference. We called variants for 2,034 samples from iHART using version GRCh37 and GRCh38 of the human reference. Human reference version had little impact on recall, but improved precision at heterozygous sites at the expense of precision at homozygous alternate sites

Figure 6 compares the variant calling performance of GATK when using human reference GRCh37 and GRCh38 on the same raw reads from the same iHART samples. At heterozygous sites, using GRCh38 greatly improves precision (median decrease in false discovery rate of 81%) at the cost of a modest decrease in recall (median increase of false negative rate of 18%). This result supports other work showing that GRCh38 improves read mapping and results in fewer false positive variant calls [18].

However at homozygous alternate sites, GRCh38 improves recall (median decrease in false negative rate of 15%) at the expense of precision (median increase in false discovery rate of 5.83x). This may be a good tradeoff since we saw in Fig. 4 that whole genome sequencing datasets typically provide very high precision at homozygous alternate sites.

### WGS datasets in low-complexity regions

Whole-genome sequencing allows us to sequence the non-coding regions of the genome along with the coding regions. The non-coding region was long thought to be “junk” DNA, but non-coding variants have recently been implicated in a variety of complex disorders [19]. However, the non-coding region of the genome contains long stretches of low-complexity regions (LCR), which can be extremely challenging to sequence using short read methods.

**Fig. 7:**
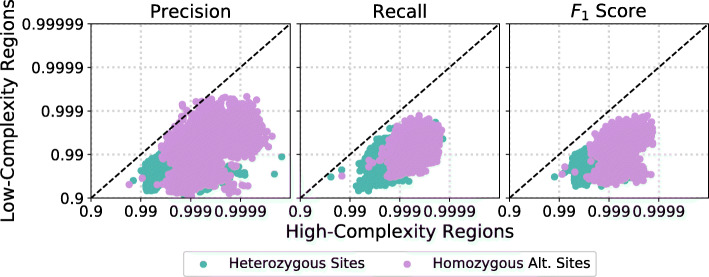
Looking at WGS data, we compare variant calling performance for low-complexity regions to all other regions of the genome (which we denote high-complexity). Precision and recall are reduced on average by 10x in low-complexity regions. However, there is quite a bit of heterogeneity across samples

We use our error-estimation method to investigate how variant calling performance deteriorates in low-complexity regions as compared to the rest of the genome, which we call high complexity regions (HCR). For this analysis we use both the iHART and SSC WGS datasets. Figure 7 shows that low complexity regions exhibit decreased precision and recall for nearly all samples. We see a median false discover rate (1-precision) increase of 5.7x and 16.6x for heterozygous and homozygous alternate calls respectively, as well as a median false negative rate (1-recall) increase of 7.1x (heterozygous sites) and 9.6x (homozygous alternate sites). These error rate increases are in line with estimates from a previous study [20]. However, this effect shows dramatic heterogeneity across samples, with some samples exhibiting nearly the same levels of precision and recall in low-complexity regions as in the rest of the genome.

### Variant calls in WES decrease in quality outside of target regions

WES is designed to target exonic regions; however, more than half of the genetic data produced by WES falls outside these target regions [21], and many of these off-target variant calls are accurate enough to be used (with imputation techniques) in association studies [22]. Furthermore, variants in these exon-flanking regions are believed to be highly relevant to disease since they may lie within promoters or UTRs which are known to impact gene expression. However in WES data, read depth decreases with distance from the target [21], likely impacting variant calling accuracy.

**Fig. 8:**
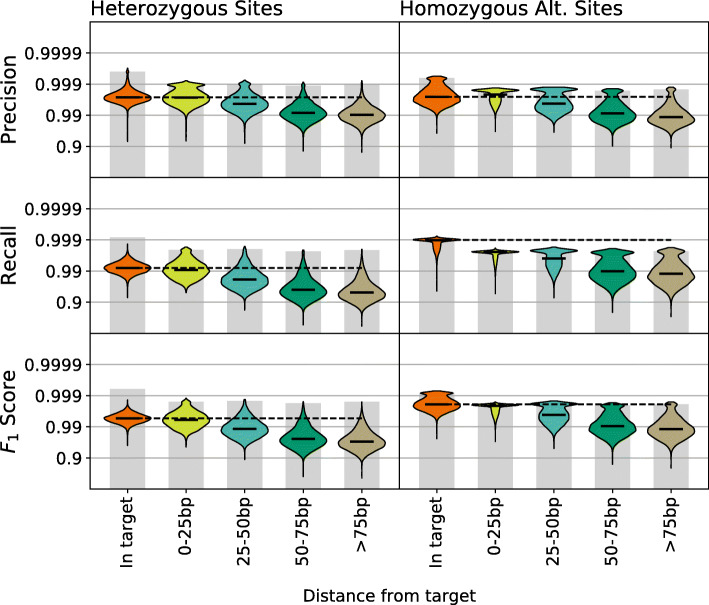
We compare variant calling performance in WES data as distance from the nearest target region increases. Performance at sites within 25bp of a target region is very similar to performance within the target regions. However, as distance from the nearest target increases, both precision and recall decay, with the most dramatic reduction occurring at sites more than 50bp from the nearest target

In order to explore how the accuracy of variant calls in WES data changes outside of target regions, we compared the calling accuracy for variants in five categories: (1) variants within the target regions defined by the exome capture, (2) variants between 0-25 bp from the nearest target region, (3) variants between 25-50 bp from the nearest target region, (4) variants 50-75 bp from the nearest target region, and (5) variants >75 bp from the nearest target region. We then estimated variant calling performance separately for each category. We found that precision and recall do in fact decrease with distance from the nearest target for both heterozygous and homozygous alternate sites (Fig. 8). While sites within 25 bp of the nearest target are nearly indistinguishable from sites within targeted regions, sites 50 bp or more away show substantially decreased variant call performance. Recall is most impacted, meaning many variants away from the targets are missed, likely due to decreased read depth.

### Variant calls from lymphoblastoid cell lines vs whole blood

Every step of the sequencing pipeline has the potential to introduce sequencing errors, including the sample preparation process. Lymphoblastoid cell lines (LCLs) are a useful tool for creating a renewable source of DNA, particularly when primary cells are in short supply. While early-passage LCLs have been shown to produce accurate genotype calls, late-passage LCLs can introduce substantial sequencing errors [13], likely due to the accumulation of de novo mutations over many cell passages. Unfortunately, when analyzing LCL-derived sequencing data, the number of LCL passages is often unknown.

**Fig. 9:**
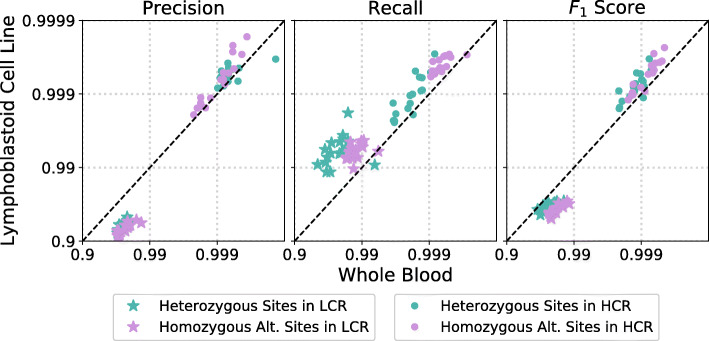
We compare variant calling performance for the same 17 individuals with samples sequenced from whole blood vs lymphoblastoid cell line (LCL). Performance in low-complexity regions (LCR) is shown with stars and performance in high-complexity regions (HCR) is shown with dots. Both precision and recall are plotted on a log-scale. Interestingly, whole blood and LCL samples exhibit very similar performance in high-complexity regions with LCLs slightly outperforming whole blood for most samples. However in low complexity regions, whole blood samples produce better precision while LCL samples produce better recall

We compared WGS error rates in LCL-derived and whole blood samples, taken from the same 17 individuals in the iHART dataset, shown in Fig. 9. The samples were sequenced at the same sequencing center and processed together using the same variant calling pipeline. We find that whole blood and LCL samples exhibit similar performance in high-complexity regions. However performance diverges in low-complexity regions, with whole blood samples producing higher precision and LCL samples producing higher recall. The lower precision of LCL samples may be due to the accumulation of de novo mutations over repeated cell passages, however it is unclear why this would occur primarily in low-complexity regions and not throughout the entire genome. Differences in the distribution of sequencing depth across the genome, observed between LCL and whole blood -derived samples [23], may also contribute to differences in variant calling performance. Overall, these results suggest that the LCL samples from the iHART dataset are faithful representations of the DNA of their donors, particularly if low-complexity regions of the genome are excluded.

### WGS outperforms microarrays at disease-associated sites

**Fig. 10:**
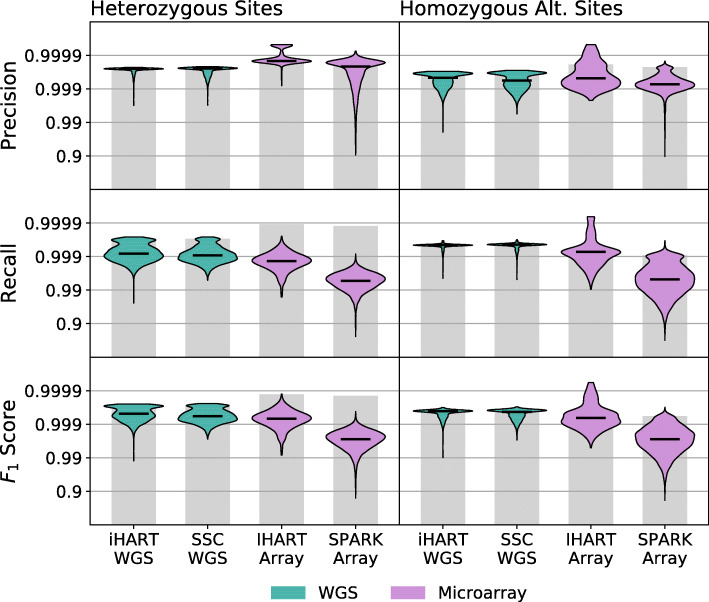
We compare the variant calling performance of WGS at sites with known disease associations recorded in GWAS Catalog to the performance of microarrays. We see that whole genome sequencing can attain precision and recall performance akin to microarrays datasets at these disease-associated sites

Only a small number of sites in the human genome have been associated with disease phenotypes. Given our previous result that WGS sequencing accuracy decreases dramatically in low complexity regions, we investigate how WGS performs at disease-associated sites. In Fig. 10, we show that WGS attains microarray-level performance for sites in GWAS Catalog. This result support the findings of previous studies which have shown that the majority of sites with disease-associations lie in regions of the genome that are easier to sequence [24].

## Discussion

We developed a method for using nuclear biological families to estimate per-sample precision and recall estimates for any sequencing platform or variant calling pipeline. We validated the accuracy of our method using identical twins. By using family structure to estimate sequencing error rates, we were able to leverage large, family-based sequencing cohorts to produce error rate estimates for thousands of individuals per sequencing method. Large cohorts allow us to produce more robust estimates of error rates and to understand how error rates vary between samples within the same sequencing dataset.

Our method can also be used to examine how much error is introduced at each step in the sequencing process. The two WGS datasets we studied demonstrated remarkably similar performance, with a median error rate on non-reference genotype calls of 0.001 for iHART and 0.004 for SSC, in line with the 0.001-0.006 range identified by another next-gen sequencing study which looked at both WGS and WES data [25]. While these datasets were sequenced on similar platforms with similar variant calling pipelines, they differ with respect to library preparation. iHART used a PCR-based protocol while SSC using a PCR-free protocol. The similar performance between the two datasets suggests that PCR does not significantly impact sequencing error. We also used our method to quantify variant calling improvements when using different versions of GATK or aligning reads to different versions of the human reference. Our approach could be used to evaluate other factors that are likely to impact variant calling performance, such as sequencing depth or read length.

By restricting our method to consider variants in certain genomic regions, we were able to replicate the results of previous studies showing that next-generation sequencing data is degraded in low complexity regions [20] and in off-target regions [21] By using large, family-based cohorts we were able to confirm these findings using much larger sample sizes than have been previously published. Both low-complexity and off-target regions often suffer from decreased read depth, so more work is needed to understand whether the increased error rates are a result of these lower read depths.

Lymphoblastoid cell lines (LCLs) are a commonly used as a renewable source of DNA. However, there have been conflicting results regarding whether LCLs may introduce substantial sequencing errors. By applying our method to iHART samples sequenced from whole blood and LCLs, we were able to show that the samples derived from LCLs demonstrate nearly equivalent error rates to the samples derived from whole blood. This supports previous results that LCLs can faithfully represent the genetic material of their donors [26]. Furthermore, our work shows how our method can be applied to any sequencing dataset containing LCL data from families to verify that error rates are within an acceptable range. This will increase confidence in the use of calls from LCL data.

Finally, we compared the performance of WGS at disease-associated sites in GWAS Catalog to the performance of microarrays and found that WGS attains microarray-level performance at these sites. These results support previous work showing that WGS can produce extremely high accuracy genotype calls [24], but care must be taken to ensure that the variant(s) of interest fall into high-confidence WGS regions.

## Conclusion

Clinical applications require reliable genotype calls, and the choice of the best sequencing platform relies on a careful understanding of each platform’s unique error profile. Genetic data from nuclear families, when utilizing the method proposed here, provides an opportunity to quantify the precision and recall of sequencing platforms and their associated software pipelines. Providing accurate error profiles for sequencing pipelines empowers clinicians to choose the best sequencing assay for each patient and to make the best-possible decisions for patient health.

## Methods

### Estimating sequencing error rates

**Fig. 11:**
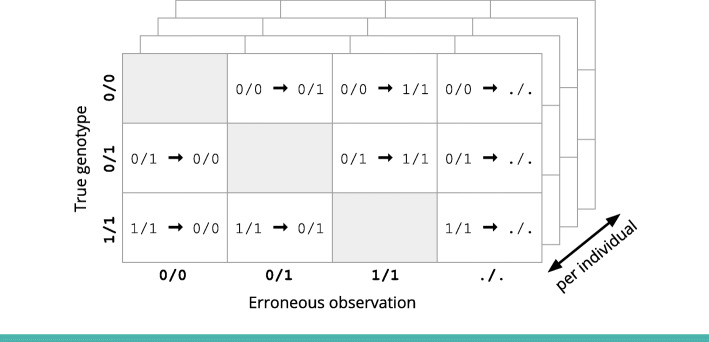
We estimate detailed error distributions for each genotype in each individual. The./. observation represents missing data

Our method estimates nine different error rates for each individual, as shown in Fig. 11. Family data allows us to detect some sequencing errors because they produce non-Mendelian observations in the family, as shown in Fig. 1. By modelling the frequency of these non-Mendelian observations, we can estimate per-individual error distributions and estimate the total number of sequencing errors in the dataset.

Let $C_{g}^{(i)}$ be a random variable representing the observed variant call for individual *i* at a biallelic site with ground-truth genotype *g*∈{0/0, 0/1, 1/1}. Sequencing errors can cause $C_{g}^{(i)} \neq g$, so our goal is to estimate the distribution of $C_{g}^{(i)}$ within a genomic dataset. Specifically, we would like to estimate $P\left (C_{g}^{(i)} = c\right)$ with *c*∈{0/0, 0/1, 1/1,./.} for all *g*, *c*, and *i*. The./. observation represents a site where the variant caller was unable to assign a genotype to the individual. By modeling these missing sites, we are able to estimate the rate of missing data for each individual while we estimate the other error rates. Here we make three main assumptions in order to simplify modelling:

1. We assume sequencing errors are rare, so $P\left (C_{g}^{(i)} \neq g\right)$ is very small.
2. We assume that all observations of Mendelian errors in a family are the result of sequencing error. This may not be true in the case of de novo variants or variants falling within inherited deletions, duplications, or other structural variants. However, we expect this assumption to hold over the majority of the genome.
3. We assume each sequencing error occurs independently in different family members, so the chance of observing multiple sequencing errors at the same site within the same family is vanishingly small. This may not be true in repetitive or otherwise hard-to-sequence regions, but we expect these special cases to be infrequent.

We define a *family genotype* as a tuple of genotypes, representing the genotypes of a mother, father, and their child(ren), respectively, at a given site. For example (0/0, 0/1, 0/1, 0/0) is a family genotype for a family of four where the mother is homozygous reference, father heterozygous, first child heterozygous, and second child homozygous reference. Some family genotypes are *valid*, meaning they contain no missing genotypes and obey Mendelian inheritance. Let $\mathcal {V}$ represent the set of valid family genotypes and let $\mathcal {W}$ represent the set of invalid family genotypes. For example, (0/0, 0/1, 0/1, 0/0) is valid. However, (0/0, 0/0, 0/1, 0/0) is invalid because both parents are homozygous reference, but one of the children has a variant.

We can represent any sequencing dataset as a set of family genotypes. Let *x*_*j*_ represent the ground-truth number of occurrences of family genotype *j*, if we could sequence perfectly without any sequencing error or missing data. We do not have access to *x*_*j*_. Instead, we have access to *y*_*j*_, the number of times we observe family genotype *j* in our dataset, in the presence of sequencing error and missing data. Since we assume that all sites obey Mendelian inheritance, for all invalid family genotypes $w \in \mathcal {W}, x_{w} = 0$. However sequencing error may cause *y*_*w*_>0.

Let *p*_*v*→*w*_ represent the probability that sequencing errors cause valid family genotype *v* to be observed as invalid family genotype *w*. We model *Y*_*w*_, a random variable representing the number of times we observe the invalid family genotype *w*, using *Y*_*w*_ to denote a random variable and lowercase *y*_*w*_ to denote a realization of that random variable (in this case, our observations). Assuming sequencing errors are rare, we can apply a generalization of Le Cam’s theorem [27] to show that the *Y*_*w*_s, as sums of multinomials, are approximately distributed as independent Poissons.

$$ Y_{w} \sim \text{Pois}\left(\sum\limits_{v \in \mathcal{V}} x_{v} p_{v \rightarrow w} \right) $$

The error of the approximation is bounded by $2\sum _{v \in \mathcal {V}} x_{v} \delta _{v}^{2}$ where *δ*_*v*_ is the probability of a sequencing error occurring at a site with family genotype *v*. Since sequencing errors are rare, we expect *δ*_*v*_ to be very small for all *v*, so the approximation is quite good.

We would like to use our Poisson approximation to develop a maximum likelihood estimate for each $P\left (C_{g}^{(i)} = c\right)$. Since we assume that the chance of multiple errors occurring at the same site within the same family is vanishingly small, *p*_*v*→*w*_≠0 only if *v* and *w* differ for only a single family member. In this case, we call *v* and *w* neighbors. Every pair of neighboring genotypes has a corresponding $P\left (C_{g}^{(i)}=c\right)$ where *i* is the index of the family member that has different genotypes in *v* and *w*, *g* is the genotype of family member *i* in *v*, and *c* is the genotype of family member *i* in *w*. For example, family genotype (0/0,0/0,0/1,0/0) has only three valid neighbors: (0/0,0/1,0/1,0/0),(0/0,0/0,0/0,0/0), and (0/1,0/0,0/1,0/0). *Y*_(0/0,0/0,0/1,0/0)_ is therefore distributed as:

$$\begin{array}{*{20}l} \text{Pois} \left(\left[\begin{array}{ccc} x_{(0/0, 0/1, 0/1, 0/0)} \\ x_{(0/0, 0/0, 0/0, 0/0)} \\ x_{(0/1, 0/0, 0/1, 0/0)} \\ \end{array}\right]^{T} \left[\begin{array}{cccc} P\left(C_{0/1}^{(0)}=0/0\right) \\ P\left(C_{0/1}^{(1)}=0/0\right) \\ P\left(C_{0/0}^{(2)}=0/1\right) \\ \end{array}\right] \right) \end{array} $$

We do not have access to *x*_*v*_, the ground truth number of occurrences of valid family genotype *v*. However, since sequencing errors are rare, we assume most valid family genotypes are observed correctly, so we can use *y*_*v*_ as an approximation of *x*_*v*_. Since our model is linear in the parameters of interest, Poisson regression will produce a maximum likelihood estimate of each $P\left (C_{g}^{(i)}=c\right)$.

### Limitations on estimating error rates in parents

Certain sequencing errors in parents will never produce an invalid family genotype. For example, if we want to understand the probability of observing a heterozygous variant call in a parent when the underlying genotype is homozygous reference, our method immediately runs up against a problem. This type of 0/0→0/1 error in a parent will never result in an invalid family call, because regardless of whether the parent is heterozygous or homozygous alternate, all of her children may inherit the reference allele. Our method therefore cannot be used to estimate 0/0→0/1 or 1/1→0/1 errors in parents. However, it can estimate these error rates for children. Throughout the paper, we report error rate distributions in children only.

### Estimating the expected numbers of errors

Once we have an estimate of the probability of a particular type of sequencing error, we can calculate the expected number of errors of this type. Let $P\left (C_{g}^{(i)}=c\right)$ represent the rate of observing variant call *c* at a site with genotype *g* in individual *i*. Let $W_{g \rightarrow c}^{(i)}$ represent the number of times individual *i* was observed to have variant call *c* at a site with true genotype *g*, then

$$\begin{array}{*{20}l} E\left[W_{g \rightarrow c}^{(i)}\right] = x_{g}^{(i)} P\left(C_{g}^{(i)}=c\right) \end{array} $$

where $x_{g}^{(i)}$ is a count of the number of sequenced sites where individual *i* has genotype *g* (for example $x_{0/0}^{(i)}$ represents the number of sites where individual *i* is homozygous reference). Our data contains sequencing errors, so we do not know $x_{g}^{(i)}$, but since we expect error rates to be small, we can use the number of times we observe individual *i* to have genotype *g* as a good estimate.

### Estimating precision and recall

Precision is the fraction of observed variants that are real, calculated with $\frac {TP}{TP+FP}$ (where *TP* represents true positives and *FP* represents false positives). Recall is the fraction of real variants that are observed, calculated with $\frac {TP}{TP+FN}$ (where *TP* represents true positives and *FN* represents false negatives). We can use these formulas along with our estimates of expected number of errors $\left (W_{g \rightarrow c}^{(i)}\right)$ to estimate precision and recall at heterozygous and homozygous alternate sites for each individual *i*.

$$\begin{array}{@{}rcl@{}} \text{Precision}^{(i)}_{0/1} &=& \frac{E\left[W_{0/1 \rightarrow 0/1}^{(i)}\right]}{\sum_{g \in G} E\left[W_{g \rightarrow 0/1}^{(i)}\right]} \\ \text{Precision}^{(i)}_{1/1} &=& \frac{E\left[W_{1/1 \rightarrow 1/1}^{(i)}\right]}{\sum_{g \in G} E\left[W_{g \rightarrow 1/1}^{(i)}\right]} \\ \text{Recall}^{(i)}_{0/1} &=& \frac{E\left[W_{0/1 \rightarrow 0/1}^{(i)}\right]}{\sum_{c \in C} E\left[W_{0/1 \rightarrow c}^{(i)}\right]} \\ \text{Recall}^{(i)}_{1/1} &=& \frac{E\left[W_{1/1 \rightarrow 1/1}^{(i)}\right]}{\sum_{c \in C} E\left[W_{1/1 \rightarrow c}^{(i)}\right]} \\ \end{array} $$

where *G*={0/0, 0/1, 1/1} is the set of possible true genotypes and *C*={0/0, 0/1, 1/1./.} is the set of possible variant call observations.

### Sequencing dataset details

Several sequencing datasets were used throughout the paper. Here we provide detailed information on the sequencing pipelines used to generate this data.

- **iHART WGS** Whole genome sequencing data from iHART [28], a dataset of multiplex autism families, containing 886 families and 3,943 individuals. Individuals were sequenced at 30x coverage using Illumina’s TruSeq Nano library kits, reads were aligned to build GRCh38 of the reference genome using bwa-mem, and variants were called using GATKv3.4. Only biallelic variants that pass GATK’s Variant Quality Score Recalibration (VQSR) were included in analysis. This dataset contains 63,206,842 informative sites, where an informative site is defined as a biallelic SNP where at least one individual in the dataset has a non-reference genotype.

- **iHART WGS v3.2** Whole genome sequencing data from iHART, sequenced as described for **iHART WGS**. Reads were aligned to build GRCh37 of the reference genome using bwa-mem, and variants were called using GATKv3.2. Only biallelic variants that pass GATK’s Variant Quality Score Recalibration (VQSR) were included in analysis. This dataset contains 75 sets of twins which are used to validate our algorithm. This dataset contains 47,890,555 informative sites.

- **iHART WGS v3.4** Whole genome sequencing data from iHART, sequenced as described for **iHART WGS**. Reads were aligned to build GRCh37 of the reference genome using bwa-mem, and variants were called using GATKv3.4. Only biallelic variants that pass GATK’s Variant Quality Score Recalibration (VQSR) were included in analysis. This dataset contains 68,963,585 informative sites.

- **iHART Array** Illumina Human BeadChip 550 microarray data from iHART containing 482 families and 2,607 individuals. This dataset contains 445,575 informative sites.

- **SSC WGS** Whole genome sequencing data from SSC [29], a dataset of simplex autism families containing 519 families and 2075 individuals. Individuals were sequenced at 30x coverage using an Illumina PCR-free library protocol, reads were aligned to build GRCh38 of the reference genome using bwa-mem version 0.7.8 and variants were called using GATKv3.5. Only biallelic variants that pass VQSR were including in analysis. This dataset contains 44,469,152 informative sites.

- **SPARK WES** Whole exome sequencing data from SPARK [30], a crowdsourced dataset of simplex and multiplex autism families, containing 5,903 families and 13,906 individuals. Exome capture was performed using VCRome. Samples were sequenced at 20x coverage. Reads were aligned to build GRCh37 of the reference genome with bwa and variants were called using both FreeBayes and GATK. Only biallelic variants within the VCRome target regions with allele quality greater than 30 were included in analysis. This dataset contains 47 sets of twins which are used to validate our algorithm. This dataset contains 2,511,175 informative sites.

- **SPARK Array** Illumina HumanCoreExome microarray data for 550K SNP sites from SPARK [31], containing 3,239 families and 13,248 individuals. This dataset contains 47 sets of twins which are used to validate our algorithm. This dataset contains 588,046 informative sites.

### Validating sequencing error rate estimates using monozygotic twins

We represent a pair of twins as individuals *A* and *B*. Let *M*_*a,b*_ be a random variable representing the number of observations where twin *A* has variant call *a* and twin *B* has variant call *b* such that *a*≠*b* and *a,b*∈{0/0, 0/1, 1/1,./.}. In order to model *M*_*a,b*_, we make a couple of assumptions:

1. We assume that the probability of observing sequencing errors in different family members at the same site is very small.
2. We assume that mismatches between twin pair *A* and *B* are caused by sequencing errors. While de novo mutations may also cause mismatches, the de novo mutation rate is around 10^−8^ per generation [32], and the current sequencing error rates are closer to 10^−5^ [20]. Thus we assume that most mismatches between twins are due to sequencing errors, not to de novo mutations.

Under these assumptions, we can model the expected number of *M*_*a,b*_ mismatches as the expected number of times twin *A* has a *b*→*a* error plus the expected number of times twin *B* has a *a*→*b* error.

$$\begin{array}{*{20}l} E\left[M_{a, b}\right] = E\left[W_{b \rightarrow a}^{(A)}\right] + E\left[W_{a \rightarrow b}^{(B)}\right] \end{array} $$

We then compare these estimates to the observed number of mismatches between twin pair *A* and *B* in our dataset. Our method relies on family data to estimate error rates, so to produce as fair a comparison as possible, we estimate error rates for each twin separately, using only non-twin family members.

### WGS variant calling in low-complexity regions

Next-gen sequencing is known to struggle in low-complexity regions (LCR). In order to examine performance in these regions as compared to the rest of the genome, we used the low-complexity regions described by [20] and generated by the mdust program. In GRCh37, 2.0% of the genome is considered low-complexity. We considered all other genomic regions to be high-complexity regions (HCR). We estimated variant calling performance in both the LCR and HCR by restricting our method to only consider variants within LCRs or HCRs respectively, using the same set of samples.

### WGS versus microarrays at disease-associated sites

To investigate performance at disease-associated SNPs, we estimated error rates for the WGS datasets restricted to sites included in GWAS Catalog [33]. We used liftover from the UCSC Genome Browser [34] to transfer GWAS Catalog sites from grch38 coordiantes to grch37 coordinates. We then compared the performance of our WGS datasets at these sites to the performance of our microarray datasets.

## Data Availability

The iHART dataset is publicly available at http://www.ihart.org/home. Approved researchers can obtain the SSC population dataset described in this study by applying at https://base.sfari.org. All code used in this paper is available at https://github.com/kpaskov/FamilySeqError. Declarations

## Abbreviations

GIAB: Genome in a bottle
GWAS: Genome-wide association study
HCR: High-complexity region
LCL: Lymphoblastoid cell line
LCR: Low-complexity region
WB: Whole-blood
WES: Whole-exome sequencing
WGS: Whole-genome sequencing
